# Urinary Angiotensinogen Could Be a Prognostic Marker of the Renoprotection of Olmesartan in Metabolic Syndrome Patients

**DOI:** 10.3390/ijms17111800

**Published:** 2016-10-27

**Authors:** Tomoko Mizushige, Hiroyuki Kobori, Hirofumi Hitomi, Yoko Nishijima, Fumihiro Tomoda, Satoshi Morimoto, Masakazu Kohno, Akira Nishiyama

**Affiliations:** 1 Department of Pharmacology, Kagawa University School of Medicine, Kagawa 761-0793, Japan; m01084tm@jichi.ac.jp (T.M.); hitomi@med.kagawa-u.ac.jp (H.H.); akira@med.kagawa-u.ac.jp (A.N.); 2 Departments of Pharmacology and of Nephrology, School of Medicine, International University of Health and Welfare, Tokyo 107-0062, Japan; 3 Departments of Physiology and of Medicine, Tulane University Health Sciences Center, New Orleans, LA 70112, USA; 4 Department of Medicine, Kagawa University School of Medicine, Kagawa 761-0793, Japan; youko.n@med.kagawa-u.ac.jp (Y.N.); mkohno@med.kagawa-u.ac.jp (M.K.); 5 The Second Department of Internal Medicine, Toyama University School of Medicine, Toyama 930-0194, Japan; tomoda@med.u-toyama.ac.jp; 6 The Second Department of Internal Medicine, Kansai Medical University, Osaka 573-1010, Japan; smorimoto@endm.twmu.ac.jp

**Keywords:** urinary angiotensinogen, metabolic syndrome, angiotensin receptor blocker, renin-angiotensin system, prognostic marker of albuminuria reduction effects

## Abstract

This study was performed to demonstrate urinary angiotensinogen as a potential prognostic marker of the albuminuria reduction effects of olmesartan in patients with metabolic syndrome. In 24 patients (eight women, 57.88 ± 2.00 years), 5–40 mg/day of olmesartan were given. Urinary concentrations of albumin and angiotensinogen (normalized by urinary concentrations of creatinine) and plasma renin activity were measured before and after the 12- and 24-week marks of olmesartan treatment. Olmesartan treatment increased plasma renin activity and decreased urinary albumin and urinary angiotensinogen significantly (*p* < 0.05). Based on the % change in urinary albumin, patients were divided into two groups, responders (<−50%) and non-responders (≥−50%), and a logistic analysis of urinary angiotensinogen before treatment showed the area under the curve as 0.694. When the cutoff value of urinary angiotensinogen before the treatment of 13.9 µg/g Cr was used, the maximum Youden index (0.500, specificity: 11/12 = 91.7% and sensitivity: 7/12 = 58.3%) was obtained. When all patients were re-divided into two groups, those with higher values of urinary angiotensinogen before the treatment (Group H, *n* = 16) and those with lower values, Group H showed significantly decreased urinary albumin (*p* < 0.05). Therefore, urinary angiotensinogen could be a prognostic marker of the albuminuria reduction effects of olmesartan in patients with metabolic syndrome.

## 1. Introduction

Obesity is a risk factor for a variety of diseases [1,2], including the development and progression of chronic kidney disease (CKD) [3]. Obesity is linked to metabolic syndrome, which is characterized by an increase in visceral fat, insulin resistance, hyperinsulinemia and dyslipidemia. Metabolic syndrome contributes to the development of type 2 diabetes, hypertension, cardiovascular disease [4] and CKD [5]. Type 2 diabetes and hypertension are widely recognized as risk factors for CKD [6]. There are many reports indicating that an increase in intrarenal angiotensinogen (AGT) and the activation of the renin-angiotensin system (RAS) are involved in CKD [7,8,9,10,11]. The intrarenal RAS is activated in obesity [3,12,13].

The angiotensin II receptor blocker (ARB) is reported to prevent the progress of renal injury in an experimental model for metabolic syndrome [14]. However, in some cases, the progress of renal injury cannot be suppressed by ARB treatments [6].

The Reduction of End points in Non-insulin-dependent diabetes with the Angiotensin II Antagonist Losartan (RENAAL) study [6] reported that treatment with the ARB losartan (50–100 mg once daily) delayed time to the first cardiovascular event, decreased cardiovascular morbidity and mortality, reduced proteinuria and decreased the rate of the progression of renal disease in patients with type 2 diabetes and nephropathy. A total of 1513 patients were enrolled in this randomized, double-blind study comparing losartan with placebo, both taken in addition to conventional antihypertensive treatment for a mean of 3.4 years. However, non-responders still exist for ARB [2].

The intrarenal RAS is regulated by multiple independent mechanisms [15]. Others and we have previously reported that the urinary AGT excretion rate could be a marker of intrarenal RAS status in hypertension [9,16,17,18], as well as in CKD [7,19,20,21,22]. However, the effect of ARB on urinary AGT has not yet been examined in patients with metabolic syndrome. Therefore, this study was performed to examine the following two hypotheses: (1) an ARB could decrease urinary excretions of albumin and AGT in patients with metabolic syndrome; and (2) urinary AGT excretion could be a prognostic marker of the albuminuria reduction effects of ARB in patients with metabolic syndrome.

## 2. Results

### 2.1. Description of the Study Population

Initially, 33 patients were recruited. Afterwards, nine patients were excluded (six participants withdrew from the study, and three participants were excluded because of incomplete samplings), and consequently, 24 patients were analyzed (Figure S1). Clinical characteristics and baseline laboratory data are summarized in Table 1 and Table 2, respectively.

### 2.2. Administration of Olmesartan

**Figure 1:**
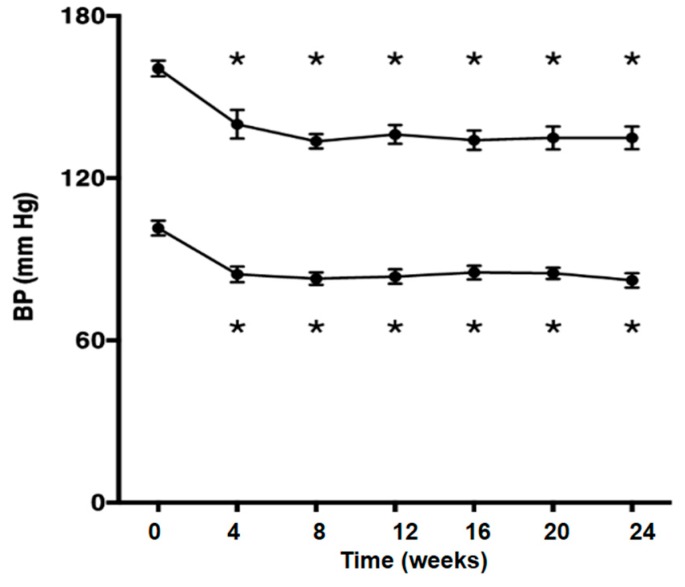
Systolic and diastolic blood pressure was decreased after the treatment with olmesartan. One-way repeated measures ANOVA followed by Tukey’s multiple comparisons test were used to compare values over the time course. *p* < 0.05 was considered as statistically significant (* *p* < 0.05).

**Figure 2:**
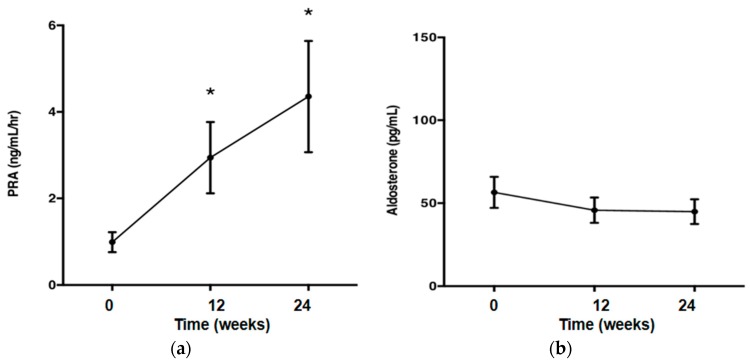
(**a**) Plasma renin activity was increased after the treatment with olmesartan; (**b**) treatment with olmesartan did not affect plasma aldosterone concentrations. One-way repeated measures ANOVA followed by Tukey’s multiple comparisons test were used to compare values over the time course. *p* < 0.05 was considered as statistically significant (* *p* < 0.05).

Olmesartan significantly decreased blood pressure (*p* < 0.05; Figure 1), but did not affect the metabolic parameters. Body weight (kg), waist circumference (cm), fasting blood sugar (FBS) and triglyceride (TG) were unchanged after treatment with olmesartan. Plasma renin activity (PRA) was increased after treatment with olmesartan (*p* < 0.05; Figure 2a). Olmesartan did not affect plasma aldosterone concentration (Figure 2b).

### 2.3. Albuminuria Reduction Effects of Olmesartan

**Figure 3:**
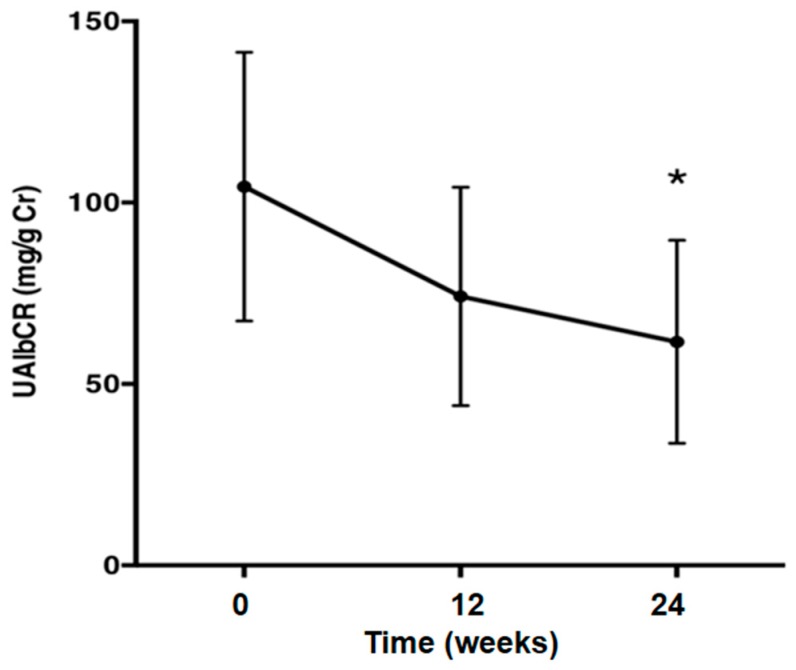
The urinary albumin/creatinine ratio was decreased after the treatment with olmesartan. One-way repeated measures ANOVA followed by Tukey’s multiple comparisons test were used to compare values over the time course. *p* < 0.05 was considered as statistically significant (* *p* < 0.05). UAlbCR, average urinary albumin/creatinine.

**Figure 4:**
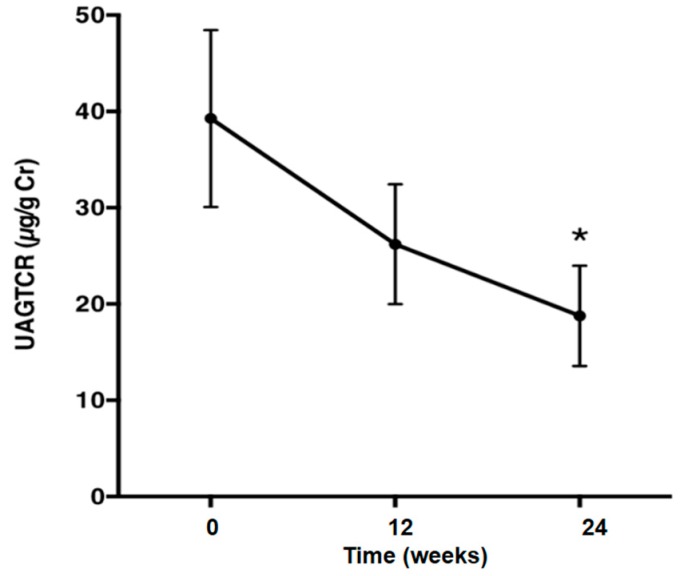
The urinary angiotensinogen/creatinine ratio was decreased after the treatment with olmesartan. One-way repeated measures ANOVA followed by Tukey’s multiple comparisons test were used to compare values over the time course. *p* < 0.05 was considered as statistically significant (* *p* < 0.05). UAGTCR, average urinary angiotensinogen (AGT)/creatinine.

**Figure 5:**
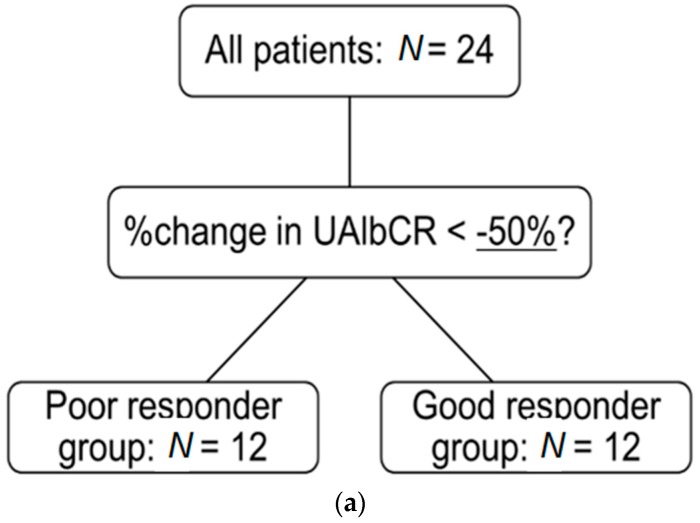

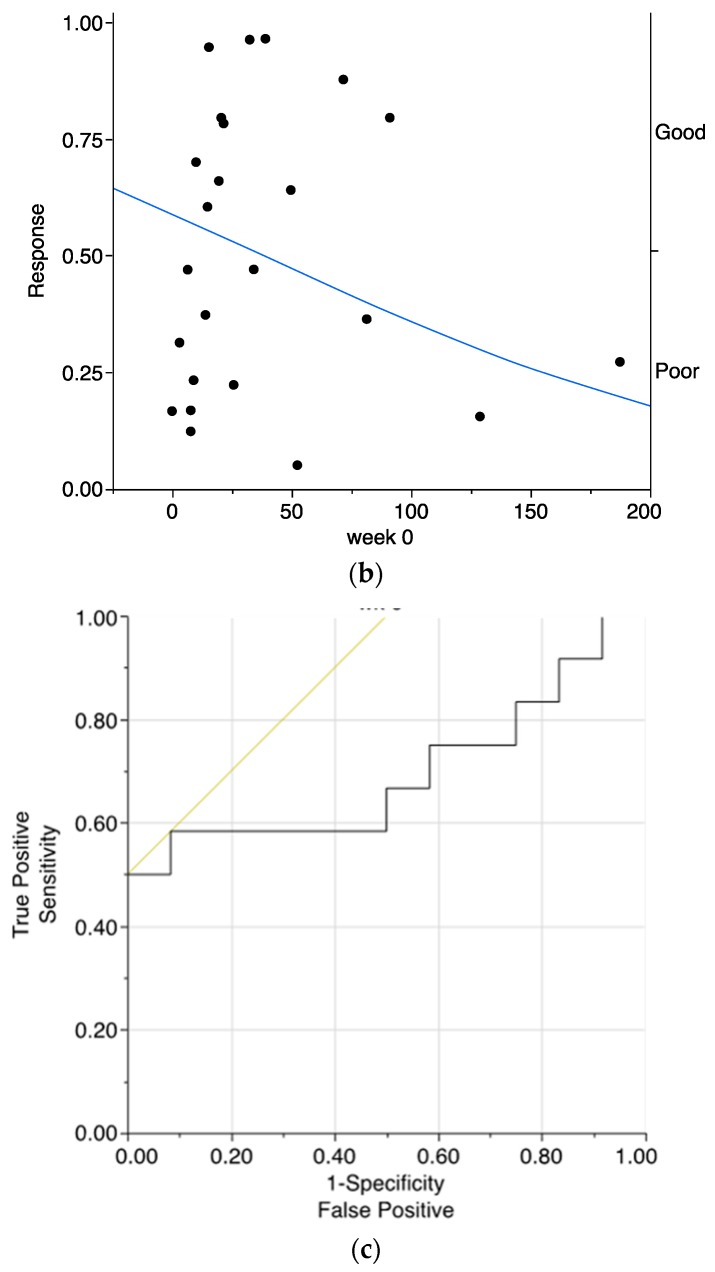
(**a**) Patients were divided into two groups based on whether or not the urinary albumin/creatinine ratio decreased less than 50%. The good responder group was defined as % change in urinary albumin/creatinine ratio <−50%; (**b**) Logistic analysis was conducted by the urinary angiotensinogen/creatinine ratio before the treatments. The receiver operating characteristic (ROC) curve was plotted. The vertical axis shows whether the urinary albumin/creatinine ratio decreased less than 50%; (**c**) Area under the curve (AUC): 0.694; cutoff value of urinary angiotensinogen/creatinine ratio: 13.9 µg/g Cr; maximum Youden index: 0.500 ((**b**,**c**) specificity: 11/12 = 91.7% and sensitivity: 07/12 = 58.3%).

The average urinary albumin/creatinine (Cr) ratio (UAlbCR) (*p* < 0.05; Figure 3) and the average urinary AGT/creatinine ratio (UAGTCR) (*p* < 0.05; Figure 4) were decreased after the 24-week treatment with olmesartan. Patients were divided into two groups based on whether or not UAlbCR decreased less than 50% (Figure 5a). The data of the clinical characteristics of the good responder group and the poor responder group at the baseline are given in the Table 3. Logistic analysis was conducted by UAGTCR before the treatments. When a receiver operating characteristic (ROC) curve was plotted, the area under the curve (AUC) was 0.694. When the cutoff value of UAGTCR before the treatments was set as 13.9 μg/g Cr, the maximum Youden index (0.500; Figure 5b,c; specificity: 11/12 = 91.7% and sensitivity: 07/12 = 58.3%) was obtained.

**Figure 6:**
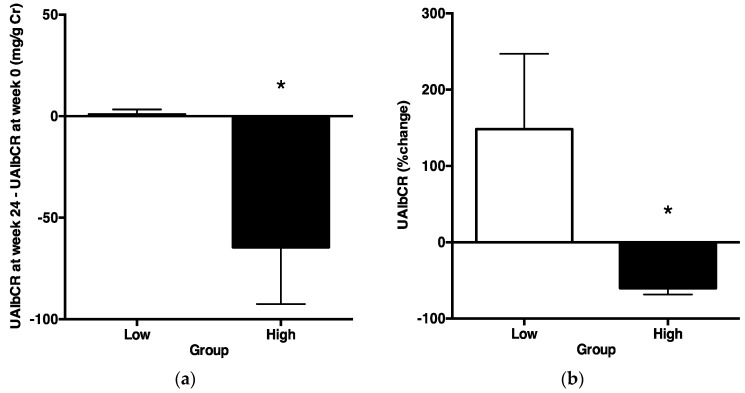
ΔUrinary albumin/creatinine ratio and % change in the urinary albumin/creatinine ratio portioned by the cutoff value of the urinary angiotensinogen/creatinine ratio before treatments. They were compared between thetwo2 groups using the unpaired *t*-test. *p* < 0.05 was considered as statistically significant. (**a**) The ΔUrinary albumin/creatinine ratio was significantly lower in Group H than in Group L; (**b**) in addition, the % change in the urinary albumin/creatinine ratio was significantly lower in Group H than in Group L (* *p* < 0.05).

Based on this cutoff value of UAGTCR at baseline, all patients were divided into two groups: the higher (Group H, *N* = 16) and the lower (Group L) group. Clinical characteristics (Table 4) and baseline laboratory data (Table 5) were not significantly different between Group H and Group L. Under these circumstances, ΔUAlbCR was significantly lower in Group H than in Group L (Figure 6a). Moreover, the % change in UAlbCR was significantly lower in Group H than in Group L (Figure 6b).

## 3. Discussion

This study was performed to demonstrate that an ARB could exert albuminuria reduction effects in patients with metabolic syndrome and that urinary AGT excretion could be a prognostic marker of the albuminuria reduction effects of ARB in patients with metabolic syndrome. Macroalbuminuria is a better risk marker than the estimated glomerular filtration rate (eGFR) in population screening of individuals who are at risk for accelerated GFR loss [23]. Microalbuminuria is widely used as a surrogate endpoint to assess renal function in patients with diabetic nephropathy [24,25,26]. The Cr and urea nitrogen of all patients at baseline are 0.72 ± 0.33 mg/dL and 13.06 ± 0.77 mg/dL, respectively, and these are normal values. Almost all of the patients in this study are in the stage of microalbuminuria. Therefore, the 24-week observation period of this study is too short to check the improvements of serum Cr, Cr clearance and eGFR. The present data demonstrated that an ARB, olmesartan, could decrease urinary excretions of albumin (Figure 3), as well as AGT (Figure 4), suggesting that an ARB has albuminuria reduction effects in patients with metabolic syndrome. Moreover, ΔUAlbCR (Figure 6a), as well as the % change in UAlbCR (Figure 6b) were significantly lower in Group H, who had a higher value of UAGTCR before the ARB treatment than in Group L, who had a lower value of UAGTCR before the ARB treatment, even though clinical characteristics (Table 4) and baseline laboratory data (Table 5) partitioned by the cutoff value of UAGTCR before the ARB treatments were equivalent between the two groups. These data suggest that urinary AGT excretion could be a prognostic marker of the albuminuria reduction effects of ARB in patients with metabolic syndrome.

There was no correlation between UAGTCR or UAlbCR and blood pressure (correlation with UAGTCR and systolic blood pressure (SBP): *R*^2^ = 0.000609, *r* = −0.0247, *p* = 0.909; correlation with UAGTCR and DBP: *R*^2^ = 0.00635, *r* = −0.0797, *p* = 0.711; correlation with UAlbCR and SBP: *R*^2^ = 0.0178, *r* = 0.133, *p* = 0.534; correlation with UAlbCR and diastolic blood pressure (DBP): *R*^2^ = 0.0114, *r* = 0.107, *p* = 0.620). These data suggest that in patients with metabolic syndrome, the albuminuria reduction effects of ARB are independent of the anti-hypertensive actions of ARB, but exert themselves via other mechanisms. Their mechanisms are the RAS/angiotensin II type I receptor pathway beyond the anti-hypertensive actions of ARB, which do not pass through the blood pressure-lowering effect of ARB. One of the possible mechanisms of CKD in metabolic syndrome patients is the activation of the RAS. Several mechanisms of the activation of the RAS are sympathetic stimulation [27], synthesis of adipocytokines in the RAS by visceral fat tissues [28] and renal hemodynamic alterations [29] despite sodium retention and clearly increased extracellular fluid volume in patients with metabolic syndrome [30,31]. The local/tissue RAS in specific tissues is associated with organ injury in the brain, heart, adrenal glands, vasculature and kidneys [15]. Kidneys have AGT [32], renin [33,34] and angiotensin-converting enzyme (ACE) [35] in their proximal and distal tubular cells. Thus, angiotensin II could be locally generated in the kidney [15]. Once angiotensin II concentration increases in the kidneys, AGT, which is the sole substrate of the RAS, will be increased further [32]. In rat glomerular cells, angiotensin II causes increasing tumor cell growth factor β1 and renal damage by hypertrophy and fibrosis [36,37]. The RAS in the kidneys is involved in the development and progression of renal injury [7,21,22]. The intrarenal RAS is activated in obesity [12,13]. This study suggests that in metabolic syndrome patients with activated intrarenal RAS, ARB has albuminuria reduction effects. Activation of the intrarenal RAS is the primary cause of renal injury in patients with metabolic syndrome [3].

There may be a correlation between UAGTCR and Cr clearance. AGT mRNA in the kidneys has been localized on the proximal tubule cells. This locally-formed and secreted AGT could dictate the intratubular angiotensin II [38,39]. It was reported that UAGTCR was correlated with mRNA and protein levels of AGT in renal tissues with IgA nephropathy [21,31]. Treatment with ARB decreased mRNA and protein levels of AGT in renal tissues and urinary AGT levels [21,40]. Urinary AGT may be unaffected by renal plasma flow or glomerular filtration rate [21]. These data suggest that the intrarenal RAS is independent of the circulating RAS and that ARB suppresses the activation of the intrarenal RAS. We previously reported that the olmesartan treatment decreased intrarenal AGT mRNA/protein levels, as well as urinary AGT levels in angiotensin II-infused rats [41]. We believe that this mechanism may account for the low levels of urinary AGT by olmesartan treatment in this study.

There were several possible explanations for how the activation of RAS in the proximal tubule cells could lead to glomerular injuries, such as albuminuria. The following three interpretations may explain this issue. First, the increase of AGT in proximal tubular cells may lead to the increase in angiotensin II in renal tissues [21], which may cause a rise of the intra-glomerular pressure and lead to glomerular injuries [7,42].

Secondly, the AGT produced in proximal tubular cells appears to be secreted directly into the apical side of the tubular lumen [43]. In addition, the AGT produced in proximal tubular cells may be secreted into the basolateral side of the tubular lumen [38]. In a study using the polar proximal tubule cell culture system, AGT was secreted into the basolateral side of the tubular lumen [44]. The secreted AGT in the interstitium may cause changes in the interstitial circumstances and, consequently, cause glomerular injury [45].

Finally, AGT was mainly expressed in proximal tubular cells in the kidney [38]. However, AGT was also expressed in glomerular cells, particularly in the mesangial cells under pathological conditions, such as diabetes [46,47,48,49]. It has been demonstrated that intrarenal AGT levels increase in diabetic rats [48,49] and humans [50] before generating renal damage. The increase in intrarenal AGT formation underlying type 2 diabetes [51,52] may be associated with the onset of diabetic nephropathy [15,46,53].

## 4. Materials and Methods

### 4.1. Participants and Protocols

This experimental protocol was approved on 29 November 2007 by the Institutional Review Board of Kagawa University School of Medicine (Kagawa, Japan, IRB: #2007CS015) and registered (UMIN 000001030). Participants with metabolic syndrome were recruited from Kagawa University, Toyama University, or Kansai Medical University from February 2008–June 2011, and written informed consent forms were obtained. Participants were patients with metabolic syndrome who met the following conditions: patients were between 20 and 70 years old, untreated by ARBs, ACE inhibitors or diuretics for the preceding 4 weeks, body mass index (BMI) ≥ 25 or (waist ≥ 85 cm (Men) or ≥ 90 cm (Women)), clinic SBP ≥ 130 mmHg or DBP ≥ 85 mmHg. In addition, at least one criterion from the following conditions was required: TG ≥ 150 mg/dL, high-density lipoprotein-cholesterol (HDLc) ≤ 40 mg/dL and/or FBS ≥ 110 mg/dL. Exclusion criteria for patients included pregnancy, treatment with ARBs, ACE inhibitors or diuretics within 4 weeks, patients with diabetes mellitus who were treated with anti-diabetic drugs, severe hypertension (SBP ≥ 160 mmHg or DBP ≥ 110 mmHg), secondary hypertension, severe renal disease (UAlbCR > 300 mg/g Cr, Cr clearance < 30 mL/min, serum Cr ≥ 2.0 mg/dL), severe hepatic disease (AST ≥ 150 IU/L or ALT ≥150 IU/L), history of major cardiac or cerebrovascular events, endocrine diseases and patients with malignancies.

### 4.2. Protocol

The participants took olmesartan 5–40 mg/day for 24 weeks. Blood pressure was checked every 4 weeks, and the amount of olmesartan was adjusted based on blood pressure (less than 130/85 mmHg and more than 100/50 mmHg). If blood pressure in patients who took 40 mg/day olmesartan over 8 weeks reached more than 150/100 mmHg, they were excluded. Blood and urine samples were collected at baseline (0 weeks) and at 12 and 24 weeks after treatment. Blood pressure was measured at baseline (0 weeks) and 4, 8, 12, 16, 20 and 24 weeks after treatment in clinic.

### 4.3. Measurements

AGT, renin activity at rest, aldosterone, FBS and TG were measured in the 0-, 12- and 24-week blood samples. Albumin, AGT and Cr were measured in the 0-, 12- and 24-week urine samples. Blood pressure and body weight were measured in the 0-, 4-, 8-, 12-, 16-, 20- and 24-week samples. Plasma and urinary AGT concentrations were measured by the human AGT ELISA kit (IBL, Gunma, Japan) [54].

### 4.4. Statistical Analysis

All statistical analyses were performed with JMP software Version 10 (SAS Institute, Inc., Tokyo, Japan) and GraphPad Prism software Version 6 (GraphPad Software, Inc., La Jolla, CA, USA). Clinical characteristics and laboratory data of all patients at baseline are reported as the mean ± S.E.M. One-way repeated measures ANOVA followed by Tukey’s multiple comparisons test were used to compare values over the time course. Logistic analysis was conducted based on whether or not UAlbCR decreased less than −50% by olmesartan treatment. The ROC curve was developed; the AUC was calculated; and the optimal cutoff value was determined. Based on this cutoff value of UAGTCR before the treatment, patients were subdivided into 2 groups. Then, the % changes in UAlbCR and ΔUAlbCR were compared between the 2 groups using the unpaired *t*-test. *p* < 0.05 was considered as statistically significant.

## 5. Conclusions

Our data demonstrate that olmesartan, an ARB, could decrease urinary excretions of albumin, as well as AGT in patients with metabolic syndrome. In addition, we show that urinary AGT excretion could be a prognostic marker of the albuminuria reduction effects of ARB in patients with metabolic syndrome. Based on the findings in this study, treatment with ARB might be recommended in untreated patients with metabolic syndrome who showed a higher value of urinary AGT before treatment.

We used only one ARB, olmesartan, but did not use multiple ARBs in this study. If we used multiple ARBs in the first study, we would not be able to judge a specific action of a specific ARB or a class-effect of ARBs. We realize that the sample size is relatively small, and there is not a control group. These are limitations of this study. Unfortunately, however, the patient recruitment in this registered clinical trial was ended, and it is not possible to increase the sample size in this study. In order to address these issues, we are now planning larger scale prospective clinical studies using multiple ARBs.

## Figures and Tables

**Table 1:** Clinical characteristics of all patients at baseline.

Parameters	Means ± SEM
Sex (Women/Men)	(8/16)
Age	57.9 ± 2.0
Height (cm)	165 ± 2
Weight (kg)	73.1 ± 2.4
BMI	26.9 ± 0.8
Waist (cm)	91.0 ± 2.0
Smoke (Yes/No)	(4/20)
Drink (Yes/No)	(13/11)
Exercises (Yes/No)	(2/22)
SBP (mmHg)	158 ± 2
DBP (mmHg)	95 ± 3
Heart Rate (beats/min)	72.7 ± 2.2

BMI: body mass index; SBP: systolic blood pressure; DBP: diastolic blood pressure; SEM: standard error of the mean.

**Table 2:** Laboratory data of all patients at baseline.

Parameters	Means ± SEM
FBS (mg/dL)	107 ± 4
Insulin (µU)	5.75 ± 0.81
HbA1c (%)	5.48 ± 0.08
TG (mg/dL)	117 ± 13
TC (mg/dL)	186 ± 6
LDLc (mg/dL)	107 ± 5
HDLc (mg/dL)	56.0 ± 3.0
AST (IU/L)	25.6 ± 2.5
ALT (IU/L)	26.5 ± 4.0
γGTP (IU/L)	61.9 ± 17.8
BUN (mg/dL)	13.1 ± 0.8
Cr (mg/dL)	0.72 ± 0.03
Uric Acid (mg/dL)	5.86 ± 0.25
PRA (ng/mL/h)	0.98 ± 0.22
Aldosterone (pg/mL)	60.8 ± 9.9
Na (mEq/L)	141 ± 0
K (mEq/L)	3.93 ± 0.05
Cl (mEq/L)	105 ± 0
White Blood Cells (/µL)	5060 ± 253
Hemoglobin (g/dL)	13.3 ± 0.3
Platelets (×10^4^/µL)	21.1 ± 1.0

FBS: fasting blood sugar; TG: triglyceride; TC: total cholesterol; LDL: low-density lipoprotein-cholesterol; HDL: high-density lipoprotein-cholesterol; AST: aspartate transaminase; ALT: alanine transaminase; γ-GTP: γ-glutamyl trans peptidase; BUN: urea nitrogen; Cr: creatinine; PRA: plasma renin activity.

**Table 3:** Clinical characteristics of the poor responder group and the good responder group at the baseline.

Parameters	Poor Responder Group	Good Responder Group	χ^2^	*p*-Values
Sex (Women/Men)	(4/8)	(4/8)	0.000	1.000
Age	55.5 ± 2.4	60.3 ± 3.2		0.244
Height (cm)	167 ± 3	163 ± 2		0.163
Weight (kg)	73.5 ± 3.7	72.8 ± 3.3		0.883
BMI	26.3 ± 1.3	27.5 ± 0.9		0.447
Waist (cm)	90.1 ± 3.0	91.9 ± 2.6		0.651
Smoke (Yes/No)	(3/9)	(1/11)	1.200	0.273
Drink (Yes/No)	(7/5)	(6/6)	0.168	0.682
Exercises (Yes/No)	(1/11)	(1/11)	0.000	1.000
SBP (mmHg)	154 ± 2	162 ± 4		0.084
DBP (mmHg)	94 ± 3	95 ± 5		0.818
Heart Rate (beats/minute)	72.0 ± 2.9	73.3 ± 3.4		0.766
UAlbCR (mg/g Cr)	139 ± 64	69 ± 38		0.356

BMI: body mass index; SBP: systolic blood pressure; DBP: diastolic blood pressure; UAlbCR: urinary albumin/creatinine ratio.

**Table 4:** Clinical characteristics of the low urinary angiotensinogen (AGT) group and the high urinary AGT group at the baseline.

Parameters	Low Group	High Group	χ^2^	*p*-Values
Sex (Women/Men)	(3/5)	(5/11)	0.094	0.760
Age	58.5 ± 3.0	57.6 ± 2.7		0.818
Height (cm)	164 ± 3	165 ± 2		0.821
Weight (kg)	73.5 ± 2.4	73.0 ± 3.4		0.903
BMI	27.3 ± 1.1	26.7 ± 1.1		0.696
Waist (cm)	91.6 ± 3.0	90.7 ± 2.6		0.813
Smoke (Yes/No)	(2/6)	(2/14)	0.600	0.439
Drink (Yes/No)	(5/3)	(8/8)	0.336	0.562
Exercises (Yes/No)	(1/7)	(1/15)	0.273	0.602
SBP (mmHg)	154 ± 4	160 ± 3		0.208
DBP (mmHg)	91 ± 4	96 ± 4		0.342
Heart Rate (beats/min)	72.0 ± 4.9	73.0 ± 2.3		0.857
UAlbCR (mg/g Cr)	8 ± 2	153 ± 52		0.0136 *

BMI: body mass index; SBP: systolic blood pressure; DBP: diastolic blood pressure; UAlbCR: urinary albumin/creatinine ratio; * *p* < 0.05.

**Table 5:** Laboratory data for the low urinary AGT group and the high urinary AGT group at the baseline.

Parameters	Low Group	High Group	*p*-Values
FBS (mg/dL)	110 ± 3	105 ± 6	0.476
Insulin (µU)	5.18 ± 1.00	6.01 ± 1.11	0.588
HbA1c (%)	5.39 ± 0.13	5.53 ± 0.10	0.408
TG (mg/dL)	115 ± 20	118 ± 16	0.912
TC (mg/dL)	188 ± 10	184 ± 7	0.758
LDLc (mg/dL)	113 ± 6	103 ± 8	0.359
HDLc (mg/dL)	53.1 ± 5.8	57.4 ± 3.5	0.541
AST (IU/L)	21.3 ± 2.1	27.8 ± 3.6	0.131
ALT (IU/L)	20.9 ± 2.3	29.4 ± 5.8	0.188
γGTP (IU/L)	31.6 ± 5.8	77.0 ± 26.1	0.108
BUN (mg/dL)	13.5 ± 1.3	12.8 ± 1.0	0.679
Cr (mg/dL)	0.68 ± 0.05	0.74 ± 0.03	0.315
Uric Acid (mg/dL)	5.48 ± 0.47	6.05 ± 0.30	0.320
PRA (ng/mL/hr)	1.14 ± 0.45	0.91 ± 0.26	0.659
Aldosterone (pg/mL)	50.8 ± 21.1	65.2 ± 11.2	0.562
Na (mEq/L)	141 ± 0	142 ± 0	0.196
K (mEq/L)	3.98 ± 0.08	3.90 ± 0.06	0.467
Cl (mEq/L)	104 ± 1	105 ± 0	0.554
White Blood Cells (/µL)	4720 ± 268	5220 ± 354	0.267
Hemoglobin (g/dL)	13.5 ± 0.4	13.2 ± 0.5	0.603
Platelets (×10^4^/µL)	22.3 ± 1.6	20.5 ± 1.4	0.402

FBS: fasting blood sugar; TG: triglyceride; TC: total cholesterol; LDL: low-density lipoprotein-cholesterol; HDL: high-density lipoprotein-cholesterol; AST: aspartate transaminase; ALT: alanine transaminase; γ-GTP: γ-glutamyl trans peptidase; BUN: urea nitrogen; Cr: creatinine; PRA: plasma renin activity.

## Supplementary Materials

Supplementary materials can be found at www.mdpi.com/1422-0067/17/11/1800/s1.
