# Supplementary material for: Urinary Angiotensinogen Could Be a Prognostic Marker of the Renoprotection of Olmesartan in Metabolic Syndrome Patients
Source: Int J Mol Sci. 2016 Oct 27;17(11):1800. doi: 10.3390/ijms17111800 (PMC5133801; doi:10.3390/ijms17111800)
Supplement: Supplementary file 1 [file ijms-17-01800-s001.pdf]

Supplementary Materials: Urinary Angiotensinogen Could Be a Prognostic Marker of Renoprotection of Olmesartan in Metabolic Syndrome Patients

Tomoko Mizushige, Hiroyuki Kobori, Hirofumi Hitomi, Yoko Nishijima, Fumihiro Tomoda, Satoshi Morimoto, Masakazu Kohno and Akira Nishiyama

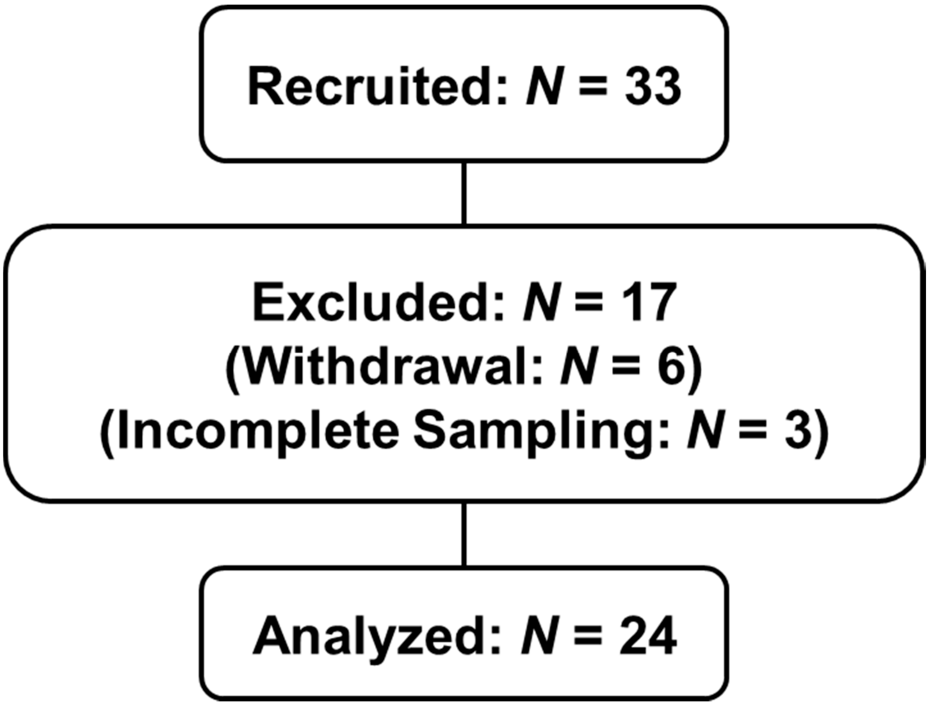

Figure S1. Trial profile: 33 patients were recruited; nine patients were excluded (six participants were excluded because of the withdrawal from this study, and three participants were excluded because of the incomplete samplings), and consequently, 24 patients were analyzed.
